# Collectanea

**Published:** 1833-04

**Authors:** 


### COLLECTANEA.

**Floriferis ut apes in saltibus omnia libant,**
**Omnia nos, itidem, depascimur aurea dicta.**

### PATHOLOGY.

### *Paralysis.* From Dr. Root's Clinical Lecture.
[*Med. and Surg. Journal.*]

I wish next to draw your attention to a case of paralysis from torpor of the nerves, apparently arising from cold. It occurred in Phœbe Munson, aged fifty, a washerwoman, who was admitted into Mary's Ward, November 1. She stated she had not been in good health for the last two years, having found, ever since that period, a gradual diminution of sensation and motion in the upper and lower extremities. Her occupation of course exposed her to cold, and, about six months ago, the loss of power and sensation had so much increased as to prevent her from any longer following it. When admitted she was very low spirited, had so little use of her arms as scarcely to be able to feed herself, and could only walk with the assistance of a person on each side of her, dragging her legs along the floor; both the upper and lower extremities were very cold, and she complained of a constant sensation of general coldness and weakness. The sensibility of the lower extremities was so much impaired, that I pinched the foot, instep, and calf of the leg, pretty severely, while talking to her, without her expressing the slightest consciousness: this was more particularly the case with the right leg; she had rather more feeling in the left. In the upper extremities, sensation was not quite so much impaired; but she was incapable of holding any thing in her hands more than a few seconds, and her grasp was exceedingly feeble. She complained of continual aching pains across the loins, occasional giddiness of the head, and dimness of vision; her tongue was white and tremulous, appetite tolerable, bowels very costive, pulse eighty, small, and compressible. Now, although she complained of this aching pain of the loins, with occasional giddiness and diminished vision, still, as I found, on examining the spine by percussion, that there was no reason to suspect inflammation or congestion of the spinal cord, that there was no heat of the head, and that the pupils dilated and contracted perfectly naturally, I was satisfied that the paralysis was dependent on a torpid state of the nerves, (probably from cold,) and determined to treat it as such.

I believe it is now pretty generally admitted, that cold is capable of producing this torpid condition of the nerves, inducing, either locally or generally, loss of voluntary power, as well as diminished sensibility. There is a curious example of this related by Dr. Clark, in the fourth volume of the Edinburgh Med. and Surg. Journal. Dr. Powell, too, published some cases of palsy, arising from this cause, in the fifth volume of the Transactions of the College of Physicians.

The indication there being to stimulate and excite the nervous system, I applied a blister to the nape of the neck, and ordered her the sixth of a grain of strychnia three times a day; and, to obviate the costiveness, gave her ten grains of the compound extract of colocynth every night; allowing her at first the house-diet, viz. meat four times a week, and soon afterwards letting her have meat daily, with a pint of porter. This treatment was continued during a greater portion of the time she remained in the hospital, the dose of the strychnia being gradually increased or diminished according to the degree of its effect. At one period, about the tenth day after she commenced it, she took half a grain three times a day, but she could not take this quantity long: the second day after taking this dose, it appeared suddenly to affect her head with giddiness, and to deprive her of all power over her limbs; but, on being put to bed, this soon subsided, and on the following day she had much more power over the voluntary muscles than she had possessed since she was admitted: indeed, on the fourth day from the commencement of her taking the strychnia, the sensibility and motive power had considerably increased; in less than a fortnight, sensation was perfectly restored, and, by the expiration of the month, she was able to walk firmly and without any support, taking each leg fairly off the ground. Ultimately, before she quitted the hospital, she was able to walk, to use her own words, "as well as ever she did in her life."

The power of the upper extremities was more slowly regained, and although it began to increase as early as that of the lower, still it was not in proportion; and, therefore, about the sixth week, I directed electric sparks to be drawn from the arms and shoulders, at the same time that she was still taking the strychnine. By degrees the power of the hands and arms returned, so that she could grasp firmly, and maintain it for a considerable time, and, when she quitted the hospital, she was quite sufficiently recovered to earn her bread. She was a woman of desponding state of mind, and would often tell me she was quite incapable of using her hands, even at the time she admitted she fed herself, laced her own stays, tied all her tapes herself, and, in fact, dressed herself entirely: so, of course, as all of you know, when a person is capable of doing this, she must have free motion and use of her arms. Last Thursday, then, she went out quite well.

You will see, by the case-book, that, when she had taken the strychnine about six weeks, some of the powdered valerian-root was ordered for her, with a drachm of the ammoniated tincture in the camphor-mixture: this was merely given her in consequence of the general feeling of coldness she still complained of. At the same time, you must not suppose I expected any great good from the valerian; the ammonia, as a stimulant, might increase the temperature of the body; the valerian, too, is unquestionably a stimulant, in consequence of the essential oil it contains, but it is contained in too small a quantity to admit of the dose in which she took it acting as such. I confess that I gave it to her with the intention of making a strong impression upon her mind, through the medium of her olfactory nerves, for, as I said before, she suffered great depression of spirits; and I believe the beneficial effects of this, as well as of most of the fœtid medicines, are to be attributed to the strong impression they make upon the mind. Afterwards, on leaving off the strychnine, when merely debility with coldness remained, I gave her some of the ammoniated tincture of iron; began first with a drachm, soon increased it to a drachm and a half, afterwards to two drachms, and then to two drachms and a half. This medicine I have always found a very excellent stimulant and tonic, and in this case it fully answered the purpose I intended. She soon now recovered her strength, the temperature of her body became natural, and she appeared to get quite fat. It is in these kind of cases, arising from cold, that strychnine is useful; but, if the paralysis arises from any pressure, or effusion, no benefit can be expected from it; or, if it should arise from inflammation or congestion, it can do no good; but, in cases similar to the one I have mentioned, from its powerful stimulating effects on the nerves, it is often useful. I have found it also beneficial in cases of paralysis arising from another cause, viz. from lead. I have a case, at the present time, in the hospital, where I can clearly trace the cause to this poison; I have given him the strychnia, and under it he is decidedly improving. I have also another case in the hospital of palsy of the wrists and fingers, but where I cannot trace the cause to the poison of lead; and therefore, as there appears to be no disease of the brain or spinal cord, I am giving him also the strychnia, and from which he is certainly deriving benefit. You know that the peculiar effect of this remedy is the production of a sensation of either pricking or involuntary contraction of the muscles, and I do not think that it is of any service in paralysis, unless one or other of these effects is produced. This, however, is not necessarily its first effect, for those gentlemen who are in the habit of going round with me will, I dare say, recollect, that many patients taking this medicine have complained of pain in the head, vertigo, and dimness of sight, and sometimes even to such an extent, that they have fallen down in a state of insensibility, without having been previously affected by any pricking or twitching of the muscles. I mention this, because Orfila and Magendie have asserted that it acts upon the spinal cord alone; the latter, indeed, has stated, that in experimenting on animals, if he divided the spinal cord at the occiput and removed it, that then the strychnia was incapable of producing any effects; but that if he left the spinal cord entire, and then removed the brain, its specific effects were produced. I am, however, satisfied, that some affection of the brain is quite as frequently produced as the tetanic affection of the muscles. It is commonly said, too, that the muscles of the paralysed limbs are chiefly affected by these convulsions, nay, that in many cases they alone are affected, the muscles of the healthy limb remaining quiet, while the paralysed limb is also said to have diaphoresis produced without the other participating. I cannot say I have found either of these to be the case. The muscles of the healthy side have been quite as much affected as those of the diseased, and when diaphoresis has been produced, I have not observed that it has been partial. Notwithstanding this, I have often seen it produce the convulsive twitching of the muscles, without the head being at all affected.

With regard to the dose of strychnia, many begin with not more than a tenth or twelfth of a grain: I rarely commence with a less dose, to an adult, than the sixth of a grain, either of the pure strychnia or of the sulphate; and, if there be no affection of the head, no pricking or twitching of the muscles, gradually increase it to a fifth, fourth, third, half, and in some instances a grain, three and four times a-day. I have never exceeded a grain for a dose: it required always to be given with caution, and to be watched. Where the dose is not increased, it appears to me to accumulate sometimes in the system; for I have known two or three instances of patients taking the same dose for two or three days without its producing the slightest apparent effect, and who have suddenly been attacked with violent twitching of the muscles, and who have fallen down in a state of insensibility. To a child, the dose I should commence with would be, at most, the twelfth of a grain, which should be very gradually increased until some such effects are produced as I have named.

---

### PRACTICAL MEDICINE.

*Convulsive Disease in Children.* Contraction may be either symptomatic or essential. In the first case, it is connected with an inflammation of the brain or spinal marrow, or their envelopes. It presents itself in the course of fevers, whether these be continued or intermittent. At the period when the two preceding cases were observed, there was an infant in the wards affected with meningo-encephalitis, which presented a permanent contraction of the left arm. M. Guersent has seen a case of a young girl, in whom there was a contraction of both arms, arising from a cerebral congestion connected with deranged menstruation. Rigid spasms of the muscles frequently show themselves in hysteria.

Essential contractions, on the contrary, appear independently of all organic lesion. They are superficial or deep. It is only to these last that the term of tetanus should be exclusively applied. These cases are attended with severe symptoms, a greater or less derangement or difficulty in deglutition, respiration, circulation of the secretions, indicating an alteration in the pharynx, heart, stomach, &c. the muscular fibres of which are the seats of tonic spasms analogous to those observed in superficial muscles. From all this it may be easily conceived how symptomatic trismus occasioned by a slight wound may cause death in less than twenty-four hours.

Superficial contractions are never accompanied with any notable derangement of the functions of digestion, respiration, and circulation. Respiration is interrupted only when the muscles concerned in this function are themselves affected. Superficial contractions are sometimes general, sometimes local. Among these last, we ought to range torticolis, cramps, tonic spasms of the muscles of the sides, &c.

Under the name of torticolis, very different diseases have been described. This affection is characterized by a permanent rigidity of one or more muscles of the neck, with an inclination of the head and an absolute impossibility of turning it. The muscles affected, the length and thickness of which diminish at the same time that they become harder, form inflexible chords beneath the teguments, which prevent the head from resuming its proper position. This affection most commonly arises from the impression of cold upon the neck. Sometimes it only remains twelve, twenty-four, or forty-eight hours; at others it assumes a chronic form. It should be distinguished from rheumatism, in which motion, though not impossible, is difficult, and attended with pain. It ought not to be confounded with articular inflammation of the first cervical vertebra, which occasions analogous symptoms.

Cramp is a contraction of the muscles of the legs and arms, and principally those of the calf. The tension of these organs takes place without any evident shortening, being attended with pain more or less acute. It is produced by the most opposite causes, lasts sometimes a few minutes, whilst at others it is protracted to eight or ten days, and even during whole months. M. Guersent has seen cases of it in children. The pain is in an inverse ratio to the duration of the disease. Very acute at first, it diminishes as the disease assumes the chronic form. M. Guersent has observed contractions of the muscles of the leg in two children of the ages of ten and twelve years, which continued for two years. One of these was attacked with croup during the continuance of the disease, and died. The autopsy was made with the greatest care, but no appreciable alteration was observed either in the brain or spinal marrow. The nerves, carefully dissected, exhibited nothing unnatural. But the muscles were in a state of hypertrophia, their pale tissue being filled with a considerable quantity of fat.

The subject of tonic spasm of the muscles of the flanks, with corresponding shortening of the parts, has given rise to an interesting treatise published by M. Thibert, which contains many cases observed in the hospital "des Enfans." Beclard has noted one case in his civil practice. A child, seven or eight years old, was suddenly seized with lameness, attended with shortening of the right inferior extremity. An intelligent surgeon, having been called in, had leeches and blisters applied, believing the case an affection of the coxo-femoral articulation. Beclard recognised a tonic spasm of the muscles of the right flank, prescribed a suitable treatment, and the child completely recovered.

Sometimes the muscular contractions of the members, affecting a great many parts, are in some degree general. Almost all the muscles of volition are liable to spasm, as was seen in the patient which formed the subject of the first case. In this instance there was immobility and stiffness of the trunk and members, as though the body was composed altogether of hard and solid parts. This contraction, although it affects a great number of parts, does not, so long as it is confined to the spinal nerves, endanger the life of the patient; but it becomes much more serious when it extends to the encephalon and ganglionic nerves, as in traumatic tetanus.

The seat and nature of this disease are altogether obscure. It does not, it is true, always terminate in death; but when it does so terminate, dissections afford no light. M. Guersent has always found the brain, spinal marrow, and nerves free from any alteration. There is nevertheless a modification of innervation, and consequently an alteration of the nerves which are the agents of this, but such alteration is not appreciable with our means of investigation. Particular cases, where the muscular tissue offers an alteration from nutrition, are to be excepted.

The etiology of essential contractions is quite obscure. These manifest themselves most frequently in weakly and puny children, and in such as are addicted to vicious habits, nervous irritations, or convulsions. Among the occasional causes, exposure to cold whilst the body is perspiring, may be enumerated as one of the most powerful. It is well known that the rigidity of the sterno-mastoid muscles is usually produced by a draught or *coup d'air*, as it is commonly called. Lastly, the presence of worms in the intestinal tube, and traumatic lesions, are regarded as causes of convulsions. Most of the causes seem to have been united in the case first cited.

*Treatment of Essential Contractions*. If an individual of a sanguineous, vigorous habit, be suddenly seized with contractions, we should not hesitate to resort to one or two general bleedings. Sauvages gives the case of a gardener, who, having entered into a pit whilst his body was covered with perspiration, was suddenly seized with a general contraction. He was at first treated for an acute pleurisy, and afterwards took diaphoretics and narcotics. He recovered in seven days. With very young, weakly, and puny, children, warm baths, vapour baths, frictions with the oil of sweet almonds, or with a liniment containing laudanum, are usually resorted to. M. Guersent thinks that opium ought not to be administered internally to children affected with contractions, except where its external use in the form of frictions is unsuccessful. Its internal use is not, however, so dangerous as has been commonly supposed.

We have seen it produce wonderful effects in some cases, and infants have occasionally taken enormous doses without showing any symptoms of congestion, the relief taking place on the first manifestion of the narcotic operation. Moisture of the skin is in general one of the most favorable signs, and this has led to the employment of diaphoretics, such as infusions of borage, the acetate of ammonia, &c. Dry frictions to the skin, vapour-baths, bags filled with warm ashes, all contribute to the same effect. Purgatives should be resorted to for the purpose of keeping the bowels open. Dr. Hamilton has recommended their use, and quoted cases showing their efficacy. Some English physicians have spoken of great advantages derived from the employment of the subcarbonate of iron, the doses of which they have carried to an enormous extent, (half an ounce daily.) It was administered unsuccessfully to the patient mentioned in the second case.

Contrivances to produce extension have been tried, and have succeeded in some instances where their employment has been seconded by the assistance of baths, emollients, &c. Extreme measures have sometimes been resorted to in some cases, such as the section of the muscles, but as this painful means was not successful, it has been entirely renounced.

For the purpose of procuring the introduction of fluids into the mouths of newly-born infants affected with trismus, it is useless and even dangerous to introduce the gum elastic cannula through the nares, it being sufficient to hold the infant in a lying position with its head strongly drawn backwards, and then introduce a teaspoon between the dental arches and the sides of the cheeks. In addition to this, baths, diaphoretics, and gentle laxatives, if constipation exist, should also be tried.—*American Journal of Med. Sciences.*

---

*Case of Spasm and Severe Pain relieved by Ligature.* Reported by J. K. Mitchell, M.D.—E. A., a female, aged seventeen, of good constitution and regular habits, resident in Sixth below Spruce, was attacked in the afternoon of the 15th of July, 1832, with slight cramp in the lower extremities, affecting chiefly the calves of the legs. The cramps increased in force as the day advanced, and in the evening a profuse alvine evacuation, of a very liquid character, was followed by nausea and pain in the abdomen. After going to bed, she vomited copiously for about half an hour, frothy, yellowish, flocculent matter of a bitter taste. At eleven P.M. the pain in the abdomen became very severe, and the intervals between the spasmodic attacks very short. The family administered laudanum freely, applied injections, sinapisms, heat, and castor-oil, without effect, and finally at half-past twelve A.M. of the 16th, sent for me.

I found her hands and feet cold and clammy, her pulse nearly natural, her countenance irregularly coloured, or flushed in ill-defined spots. Incessant motion and mournful cries expressed the severity of her pain. No evacuation of any kind had occurred after eleven o'clock, nor had there been any cessation of pain.

Bled her thirty-six ounces, administered laudanum and castor oil, ordered enemata, sinapisms, and dry heat with frictions. At half-past one, no abatement of symptoms. Applied a tourniquet round the middle of the forearm so tightly as to demand nearly all my strength in turning the key. An immediate removal of pain and nausea ensued; the patient lost the irregular flush, the extremities became warmer, and every morbid symptom disappeared. To try the effect, the tourniquet was loosened, and the pain immediately recurred. It was then kept tight for an hour, when it was relaxed without inconvenience. Soon after I left the patient, the pain and nausea returned, and the nurse endeavoured in vain to check them by the tourniquet. At half-past five A.M. I found the case as at eleven P.M., and, on tightening the tourniquet, which was badly screwed up, I again succeeded in suppressing the symptoms. At this visit I sent for my late pupil, Dr. Smiley; and, after relaxing and tightening it, the tourniquet demonstrated the perfect control in which the pain and nausea were held.

Ordered a pill of Ol. Croton, gtt. j., Tart. Emet. gr. $\frac{1}{6}$th, Calomel, gr. ij., Rhei Pulv. grs. iv., to be repeated every hour until effective. At ten A.M. found that five pills had been taken, and an alvine evacuation of black fetid matter, of the consistency of tar, had been passed.

At twelve o'clock Dr. T. Harris saw the case with me. At this visit the nausea returning, it was instantly checked by tightening the tourniquet.

Once in the course of the day the pain returned slightly, and the vomiting recurred twice. In every instance the symptoms were removed by the tourniquet.

17th, four P. M. The case is apparently convalescent.

*Remarks.* The remarkable effect of the tourniquet in so severe a case, where ordinary measures for cure, vigorously applied, totally failed to abate a single symptom or to allay a single pang, renders this remedy worthy of further trial. Hitherto experiments with it have not been made extensively enough to fix its relative value. The objections of the patient have commonly prevailed upon the physician from making it tight enough, and the practitioner has been deterred by the dread of sphacelation. Neither should be regarded, because the patient approves when he observes the beneficial result, and the circulation cannot be entirely checked in the interosseous vessels of the forearm. The beneficial effect is only complete when the hand is brought to a close resemblance to that of a patient under cholera asphyxia.

In the present case the tourniquet remained tight for four successive hours without the slightest subsequent disadvantage. In cases of great severity, the screw should be turned until the good effect is obtained, for it is almost certain to follow an adequate application.

This remedy, originally Japanese, travelled through China to the Russians, and was brought with the cholera into Europe, where it was occasionally approved in practice.

It is to be observed that, for a cure, ordinary medical means must be employed, for the tourniquet only holds pain and spasm in temporary check.—*Ibid.*

*Two Cases of Accidents, from Admission of Air into the Veins during Surgical Operations.* By John C. Warren, m.d. Professor of Anatomy and Surgery in Harvard University. Some professional men have expressed doubts as to the accidental admission of air into the veins during surgical operations. Such doubts appeared well founded when the occurrence first attracted the attention of surgeons; especially on considering that veins about the neck were so very often wounded in the removal of tumors; and that some of them, as the external jugular, are frequently opened for the purpose of taking blood, without any unfavorable consequences.

Not long since I had evidence of the existence of such cases in two of my own patients, within no great distance of time from each other.

Case I. Mr. William Burrill, of Salem, aged sixty, was admitted into the Massachusetts General Hospital, on the 16th October, 1830. He had a cancerous affection of the left side of the face and neck, of the extent of three or four inches diameter. It was hard at the edges, of a livid red colour, ulcerated in the centre, very offensive, very painful, and had made an impression on the general health. The parotid gland, the submaxillary, the sublingual, and all the textures excepting the bone, were involved in the complaint. The lower jaw itself was thought to be diseased at first, but it afterwards appeared that it was not so. In so bad a state of things, I felt very little hope of being able to eradicate the disease, and would not have attempted any operation, had not the patient solicited it.

Considering the extent of the disease, that important blood-vessels would be divided, namely, the facial and sublingual arteries, probably the temporal, and even the external carotid, I thought it best to begin by securing the carotid trunk. An incision for this purpose was begun opposite the thyroid cartilage, and carried two inches downwards. The platysma muscle was divided; the edge of the mastoid exposed and dissected. Thus far, only a few drops of blood were discharged. The face of the sheath of the great vessels was a little uncovered, when a small effusion of venous blood appeared under the knife, and checked the operation. At that instant a very distinct sound was heard, like the passage of air through water. A few bubbles were seen in the venous blood, the flow of which was immediately arrested by applying a finger on the part. The patient exclaimed, "I am faint." On regarding his countenance, it was not pale, but livid, almost black, and the muscles agitated by a convulsive motion. The respiration became deep, laboured, and stertorous, like that of apoplexy. Committing the impression of the vein to Dr. Haward, who assisted me, I examined the pulse at the wrist, found it distinct, but very slow. The wound not bleeding, and very little blood having been lost, I directly opened the temporal artery, and the blood poured from it with great freedom. As it flowed, the respiration became more frequent and less laborious; the pulse at the wrist more natural. The leaden colour in the cheeks assumed a reddish tinge; and the alarming character of the symptoms was evidently diminished. About twenty minutes elapsed during these changes. At the end of half an hour I judged it safe to remove the patient to his bed, where he lay in a state of insensibility for two hours; at the expiration of which he awaked as from sleep, still breathing like an apoplectic. The night was passed without any accident, and on the following morning he was as well as usual, with the exception of a moderate soreness over the thorax, and a headach.

In seven days after the accident described above, the operation was performed without tying the carotid artery.

The diseased parts were included in an elliptical incision, extended from the lobe of the ear to the upper part of the neck, and including the submaxillary, the sublingual, and parotid glands, all of them in a morbid and disorganized state. The os maxillare inferius was not diseased. The hemorrhage was copious: but readily arrested, with the exception of that from a large vein, which, from its depth under the jaw, could not be distinguished so as to admit the application of a ligature, and was therefore compressed by a sponge. The veins below the wound were compressed by Dr. Hayward during this operation. The patient experienced a slight faintness, which soon passed off. He had no bad symptoms, and on the 10th of December, the wound being nearly healed, he requested his discharge; which was granted.

Case II. Nancy Bunker, of Trenton, in Maine, married, her age thirty-three. Three years since, she noticed a hardness in the right breast, which increased till it involved the whole gland in a tumor, very hard, moveable, yet obviously connected with the pectoral muscle by a morbid adhesion. The nipple is drawn in. The axilla is occupied by a considerable tumor, of a globular form, and quite hard. The disease has been accompanied, during the last year, with very constant lancinating pains. The patient is desirous of an operation; has a strong conviction that she shall not recover, yet is perfectly tranquil and resigned.

*Operation.* The patient sat in a chair. The right arm was extended, raised above a horizontal line, in order to give tension to the skin, and permit access to the armpit, and was supported in this position by an assistant. The skin on the surface of the breast, with the diseased nipple, were included in an oval incision; the breast was dissected from the pectoral muscle, and left connected with the axillary glands, while the extirpation of these glands was effected. As they adhered to the great axillary vessels, they were cautiously detached by dissection, and by insinuating the finger where the cellular substance was loose, between the tumor and the great vein. This separation was nearly effected, only a slight connexion still existing at either extremity of the tumor. Proceeding to separate it, at the outer part of the axilla, a vein was divided, and a small quantity of venous blood discharged. This obscured the parts at that point, and the knife was therefore carried to the other extremity of the tumor. Scarcely was this done, when the patient struggled, and, on regarding her, I perceived her complexion to be a livid pale colour; and at the same instant the bubbling or clucking noise was heard, though indistinctly, but the place from which it issued was not visible, the surrounding skin and fat having fallen over it at the moment of the transfer of the knife. Directly the axilla was compressed, the patient became insensible, breathing in a distressed manner, as in apoplexy. The tumor was at once separated. The posture of the patient was changed, and she was supported by those around. Some brandy was poured down, and ammonia was introduced into the nostrils; the pulse, however, became less distinct every instant. Cloths dipped in hot water were thrown over the extremities; strong frictions were applied to the chest and to all parts of the body; considerable quantities of brandy were again poured down the throat. At this moment the livid colour of the cheeks gave place to a suffusion of vermilion red, and no glow in the cheek of a youthful beauty ever gave one so much pleasure as that flush. I was turning to the class, who watched the different operations with intense anxiety, to say, "the danger is over!" but checked myself, and continued the efforts. But the flush soon passed off; the lividity reappeared; the respiration became more feeble; pulse at the wrist scarcely perceptible; and, notwithstanding the redoubled applications of external heat and moisture, the extremities and whole body cooled rapidly, and presently the respiration ceased.

As a last effort, I opened the larynx and put in operation the inflation of the lungs by a bellows, in a very speedy and perfect manner, imitating the movements of inspiration and expiration with great exactness, continuing the general application of heat and frictions to the whole surface. These administrations were continued for about twenty minutes longer, without any encouraging appearances. At the end of this time, I perceived there was no remaining hope of the restoration of my patient to life. The friends being anxious to take advantage of a vessel then sailing for their home, the body was soon after removed, and no opportunity afforded for examination.

The effects of the entrance of air into the blood-vessels appear to have been known to Lieutaud, Morgagni, and other distinguished pathologists; but the danger of such an occurrence in surgical operations does not seem to have been adverted to, until the operation of M. Dupuytren, in which the admission of air through the external jugular proved suddenly fatal. Since the publication of this fact, the occurrence has presented itself to many surgeons in Great Britain and this country.

A natural scepticism in regard to these accidents has arisen from not considering the peculiar action of the auricles of the heart. How, it is asked, can air force itself into the veins, which are already filled with blood, and at the moment this fluid is discharging itself from an aperture in the vessel! The possibility of the accident will however be admitted, on recollecting that the auricles act not only like an expelling syringe, when they drive the blood into the ventricles, but that they have the power of suction when they dilate themselves, thus sucking the blood from the two cavæ, and, of consequence, from the great veins connected with the cavæ. This suction-power of the auricles explains what would otherwise be unintelligible, the movement of blood through the large inactive veins near the heart.

There remains another difficulty. Why do not the sides of these veins collapse when the blood is pumped from them by the auricle? and if they do thus collapse, how can air be drawn in through a small aperture in one of these vessels? This objection has been removed by M. Bèrard, who has shown that the large veins near the heart are protected by fasciæ, connected to the coats of the veins by cellular substance. The fasciæ themselves are attached to bones, in such a way as to prevent their collapsing on the veins. Further, it may sometimes happen that the coats of a vein assume a morbid structure, which gives them an unhealthy rigidity, and in this manner prevents their collapse. This occurred to M. Dupuytren, as I am informed by my friend Dr. Lodge, who was in Paris at the time. M. Dupuytren, being about to divide a large varicose saphena vein, expressed some apprehension that air might be admitted, and that the result would be fatal. The vein was divided, the peculiar sound of the entrance of air was heard, and the patient expired.

In the first of the cases above related, the vein opened was a small vein crossing the neck from the median external jugular to the great internal jugular. At least I presume this to have been the vessel; though there can be no certainty of its identity, the incision in the neck being small, the parts not much uncovered, and the sheath of the internal jugular not opened. This small vein, stretched across the neck, was kept tense by its attachment to fixed veins at each extremity, and would thus be in a favorable position for the admission of air on the suction of the auricle.

The vein opened in the second case was the subscapular. It did not seem to be large, though perfectly visible before it was cut, and the point of the incision was at a sensible distance from the great axillary vein, say nearly an inch. The dissection had separated it from the surrounding parts in a considerable degree. The axillary cavity was extremely dissected; so that the attachments of the fasciæ covering the great vein must have been much relaxed.

Here then was a small vein, at some distance from the heart, dissected from the surrounding parts; and its receiving vein also dissected. The coats of the vein were not visibly diseased. The explanations of M. Bèrard will not therefore apply. The cause of the phenomenon in this case is to be sought in the position of the arm. The limb was extended and elevated; in consequence of which, the axillary vein was in a state of extreme tension. The subscapular vein was also kept tense by the chain of axillary glands and by the weight of the depending breast; for this organ had not been separated from the glands, in order that they might be drawn down by it and exposed.

The possibility of these accidents under circumstances like those above, where there could be so little reason to fear them, must be a cause of anxiety to operators, in the removal of tumors from the neck and armpit; and I know of no effectual means to guard against them. *Pressure* on the vessels intervening between the disease and the heart would often be impracticable; and, where it could be applied, the tension of the fasciæ would generally render it abortive. Causing the patient to *expire* the air from the lungs could only be practised for a moment. *Change of position*, by relaxing the vessels, would do something; yet the state of tension must in many cases be resumed, in order to carry on the operation. The immediate compression of the vessel on the appearance of the accident, might sometimes save the patient from death, though not from very threatening appearances: for, in the first case, the patient's life was preserved; but, although the accident was instantaneously arrested, he was saved with difficulty.

On a view of all these considerations, it appears prudent to suspend an operation in the vicinity of the heart, at the instant of the appearance of venous blood from a suspicious point; and to compress the vessel, that time may be had for observing whether dangerous symptoms are likely to arise; and if these actually appear, we should directly resort to the means spoken of: First, compress the orifice of the bleeding vein with the utmost care; second, apply pressure on the veins between the wound and the heart; third, relax the part in which the vein is seated; fourth, the patient may be directed to expire the air from his lungs.

The means to be pursued for saving life, after air has been admitted, have been stated in the history of these two cases, and I know of none more effectual than were adopted. The opening of the temporal artery gave great relief to Case I.; it was not resorted to in Case II., because the patient had already lost as much blood as she could spare during the operation.—*Ibid.*

*** For further remarks upon this interesting subject, and for additional cases and some experiments, showing the fatal effects which may arise from the admission of air into the veins during surgical operations, we refer our readers to our Journal for February 1831, pp. 122 and 175.—Editors, *London Med. and Phys. Journal.*

